# Highly Enhanced Inductance Sensing Performance of Dual-Quartz Crystal Converter

**DOI:** 10.3390/s19092188

**Published:** 2019-05-11

**Authors:** Vojko Matko, Miro Milanovic

**Affiliations:** Faculty of Electrical Engineering and Computer Science, University of Maribor, Koroška cesta 046, 2000 Maribor, Slovenia; miro.milanovic@um.si

**Keywords:** inductance-to-frequency converter, dual quartz crystal operation, enhanced inductance sensing performance

## Abstract

This paper presents ways of inductance sensitivity improvement in a quartz crystal converter for low inductance measurement. To improve the converter’s sensitivity, two quartz crystals that were connected in parallel and additional capacitance connected to the two quartz crystals in the oscillator’s circuit are used. The new approach uses a converter with special switchable oscillator and multiplexer switches to compensate for the crystal’s natural temperature-frequency characteristics and any other influences, such as parasitic capacitances and parasitic inductances, which reduce them to a minimum. The experimental results demonstrate improved sensitivity and well-compensated dynamic temperature influence on the converter’s output frequency. The fundamental quartz crystal frequency-temperature characteristics in the temperature range between 0–40 °C are simultaneously compensated. Furthermore, the converter enables the measurement of the influence of its own hysteresis at different values of inductances at the selected sensitivity by parallel capacitances connected either to the single- or dual-quartz crystal unit. The results show that the converter converting inductances in the range between 85–100 μH to a frequency range between 1–150 kHz only has ±0.05 ppm frequency instability (during the temperature change between 0–40 °C), which gives the converter a resolution of 1 pH. As a result, the converter can be applied where low inductance measurement, nondestructive testing, impedance change measurement, and magnetic material properties measurement are important.

## 1. Introduction

High inductance sensing performances are demanded in various areas, such as magnetic material properties measurements, medical and pharmaceutical measurements, and physical measurements. High sensitivity and resolution are, for instance, provided by the magnetoelectric (ME) sensors (including piezoelectric magnetostrictive sensors), which directly convert the magnetic field into an electrical signal, allowing for measurements of low intensity magnetic fields at low frequency. The critical aspect is an encapsulation process, because the used magnetostrictive material is temperature sensitive [1,2]. Inductance converters, on the other hand, are used in many applications for the measurement of inductances and magnetic material properties, for the biomagnetic signal detection, detection of magnetic beads, detection of microscale magnetic particles from macroscale materials, etc. [3,4,5]. The measured physical parameters are correlated to the material’s composition or measured physical characteristics through the known relation between physical parameters and chemical properties to measure magnetic material properties. To obtain high-quality measurement results in the above specific cases, the high-sensitivity detection of low inductance and/or low magnetic field changes has to be coupled with high-precision frequency stability measurement.

Recently, numerous studies have investigated inductive sensors. The development of passive measurement technology can address issues, such as temperature influence, which is particularly problematic in environments that involve higher temperature. As these sensors exhibit small (but non-negligible) temperature drift, temperature compensation is crucial in obtaining accurate measurement data. However, the lack of the measurement accuracy in environments that involve changing temperature is often a problem in these sensors [6]. One of the research works studied the effect of relative movement on the inductive sensors to improve the sensitivity of inductive sensors in ferromagnetic particle detection. The study has shown that the velocity of this particle improves the sensitivity of the inductive sensor [7]. The next study reports of an ac magnetic field sensor with pT/√Hz sensitivity (around 500 Hz) based on self-biased magneto-electric composites made using piezoelectric Pb(Mg_1/3_Nb_2/3_)O_3_-PbZrO_3_-PbTiO_3_(PMN-PZT) single crystals in macro-fiber form and a magnetostrictive Ni plate. The loss factor of the piezoelectric layer was one of the key parameters that affected the magnetic field sensitivity. Helmholtz coils and a lock-in amplifier were used to characterise the sensitivity of the ME composite magnetic sensor [8]. In order to improve the sensitivity of the sensor, a two-dimensional electron gas (2DEG) AlGaAs-InGaAs-GaAs Hall device, which was designed and optimised with respect to the measurements of low magnetic fields is reported. This device has been fully characterised with respect to noise and sensitivity measurement, and applied in low magnetic field detection (100 nT–100 µT) [9]. Magnetoelastic sensing technology has also made significant progress over the last ten years. The magnetoelastic sensor was inserted inside an inductive solenoid in one of the magnetoelastic methods, and the impedance (with inductance *L*_s_ ~ 100 μH) of the solenoid was measured as a function of the resonant frequency. The sensitivity of this method was only improved with new sensitive magnetoelastic materials [10,11,12]. A piezoelectric quartz crystal resonator (PQCR) in series represents yet another approach to inductance sensitivity improvement, with an inductor being suggested as sensitive inductance-to-frequency transducer circuit. The operating principle underlying this work is based on phase lock oscillator detection of tiny changes in inductance of the sensing coil. However, this method does not analyze the temperature influence on the measurement error. [13]. The inductance measurement method, as a way of improving frequency pullability in the quartz oscillator, was already used in the past to compensate for quartz crystal stray capacitances. To this end, a special AT-cut fundamental quartz crystal working near the antiresonance frequency was selected. The magnetic sensing of the circuit was highly improved by modifying its equivalent circuit with load inductance and series tuning capacitance. The method researches the sensitivity improvement, however, it does not simultaneously address the issue of the temperature compensation of the quartz crystal [14]. 

However, this paper presents different ways of inductance sensitivity improvement in quartz-crystal converters, such as the enhancement of the inductance-to-frequency sensitivity using dual-quartz crystal unit. Converter’s inductive sensitivity is improved in two different ways. In the first way, an additional quartz crystal unit is connected in parallel to the first one. In the second, parallel connection of the capacitors and series inductance to the dual-quartz unit is used. Additionally, analogue multiplexers and an I/O control device are used, which enable software-controlled changing of inductance and capacitance in the converter circuit as well as oscillator’s frequency. This approach allows for the checking of the converter’s own inductance-to-frequency characteristics at different sensitivities and an analysis of its hysteresis, which plays a vital role in low inductance measurement. With an additional reference oscillator, higher frequencies of the oscillator can be transformed into a lower frequency range that is more convenient for further signal processing. By switching between the reference and measuring inductance, the quartz crystal non-linear self-temperature frequency characteristics as well as parasitic influences of the converter electronic circuit are compensated. An additional advantage of the proposed method—when comparing the temperature stability during the temperature changes in the range between 0–40 °C to other methods [15,16,17]—is that no temperature sensor is needed. 

Moreover, a comparison to other methods, such as the Colpitts circuit, reveals that the latter does not have temperature compensation. The Balance bridge circuit with two sensing inductances has a better temperature stability than the Colpitts circuit and the Phase circuit—a pulse width modulation circuit allows for the optimization of temperature stability, but does not offer as good temperature compensation as the proposed method [18,19]. The sensor methods that are based on the principle of the influence of the quartz crystal on the resonator’s equivalent circuit display a temperature drift, despite the high frequency stability of the oscillator’s resonant frequency. However, this drift can be compensated by the method that is suggested in this paper without any additional temperature stabilization of the converter’s environment or additional temperature measurement using temperature sensor [20].

## 2. Experimental Inductance-to-Frequency Converter

### 2.1. Switching Between the Measuring and Reference Impedance

**Figure 1:**
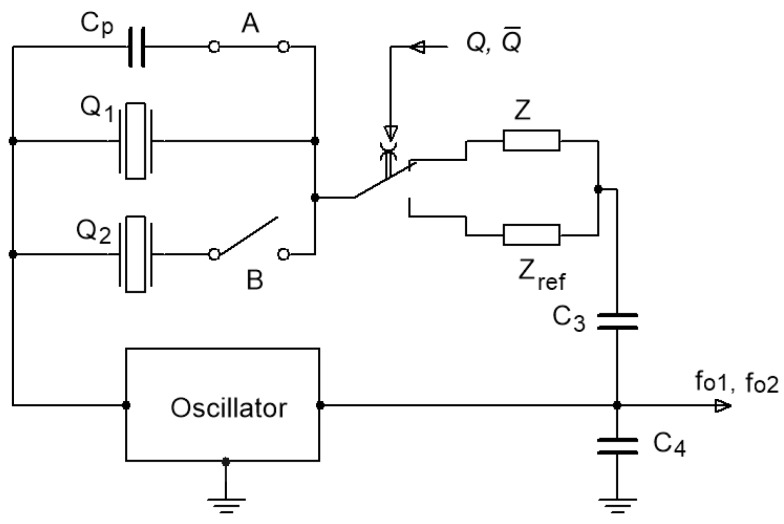
Oscillator’s switching principle.

The basic idea of this new approach for the quartz inductance-to-frequency converter lies in the switching between the reference impedance *Z*_ref_ and converting impedance *Z* and is connected in series with two quartz crystals with the equal frequency (connected in parallel) (Figure 1) and in the ways of sensing performance improvement when compared to one quartz crystal. Two different frequencies *f*_01_ and *f*_02_ are produced at the output through the switching of the two different impedances within a few seconds. These two frequencies depend on quartz crystal’s (*Q*_1_) serial resonance frequency and serial influence of both impedances *Z* and *Z*_ref_ (Figure 1). 

In both cases, the influence on both frequencies is the same due to the temperature-frequency dependence of the quartz crystal and the temperature influence of both impedances, provided that these two impedances are inductive in character. The temperature influence can be compensated (reduced to a minimum) through further processing of both frequency signals and formation of the frequency difference between them. Moreover, any other additional influence on the oscillator’s frequency, such as parasitic capacitances, inductances, and ageing of the elements is reduced, since their influence on both frequencies is the same. Figure 1 shows the principle of the oscillator circuit switching (by signals Q and $\bar{Q}$, which are digital signals 1 or 0) between the reference impedance *Z*_ref_ and the converting impedance *Z*. By switching the switch A the capacitance *C_p_* is switched in parallel to *Q*_1_ to further increase the frequency pulling range. With the switch B, the second crystal is connected in parallel to the first one to additionally increase the frequency pulling range.

### 2.2. Electrical Equivalent Circuits for Single- and Dual-Quartz Crystal Unit Operation

**Figure 2:**
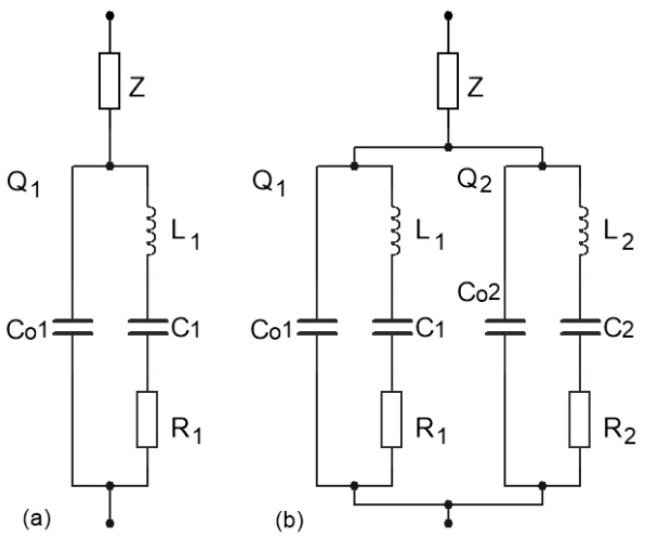
(**a**) Equivalent single-quartz crystal electrical circuit and (**b**) additional connection of a second crystal in parallel to the first one. In both cases, the same impedance *Z* is connected in series.

Through impedance *Z* (Figure 2) connected in series with the quartz crystal, the crystal’s serial resonance frequency in a given range can be changed, thereby also changing the oscillator’s frequency in the oscillator. Figure 2a shows a single-quartz crystal equivalent electrical circuit, together with impedance *Z*. The capacitance *C*_01_ is real capacitance, which comprises the capacitance between the quartz electrodes and the stray capacitance that are associated with the mounting structure. The other components represent the crystal in an operational or motional state: *L*_1_, *C*_1_, and *R*_1_ represent the “motional inductance”, the “motional capacitance”, and the “motional resistance”, respectively. Figure 2b illustrates a case where an additional quartz crystal that is connected in parallel is added to the first one to further increase the changing of the serial resonance frequency (frequency pulling). 

The impedance of the single quartz crystal with impedance *Z* in series (Figure 2a) is explained by Equation (1) in case *Z* is inductance *Z* = j*ωL*_L_ + *R*_L_. In the case of a solenoid, the inductance *L_L_* = *μ*_0_*μ_r_N*^2^*A*/*l* (*μ*_0_—the permeability of vacuum, *μ_r_*—the relative permeability inside of solenoid, *N*—the number of turns, *A*—the cross-sectional area, *l*—the length of solenoid). 

The frequency ratio Ω = ω/ω_o_ is determined near the crystal’s (Q_1_) serial resonance frequency, where $\omega_{0}=1/\sqrt{L_{1}\cdot C_{1}}$ is the series resonant frequency of Q_1_. Frequency ratio Ω describes the impedance change near the Q_1_ resonance frequency in the range of change Ω = 0.998–1.038, whereby Equation (1) describes the complex impedance [21,22,23].

(1) $$\bar{Z_{1}}\left(\Omega \right)=R_{1}\frac{1+j\frac{\omega_{0}L_{1}}{R_{1}}\left(\Omega -\frac{1}{\Omega }\right)}{1+\frac{C_{01}}{C_{1}}\left(1-\Omega^{2}\right)+j\frac{C_{01}}{C_{1}}\cdot {\frac{R_{1}}{\omega_{0}L}}_{1}\cdot \Omega }+j\frac{\Omega \cdot L_{L}}{\sqrt{L_{1}\cdot C_{1}}}+R_{L}$$

In the case of two crystals with equal frequency (*L*_1_ ≅ *L*_2_ and *C*_1_ ≅ *C*_2_ (Figure 2b) connected in parallel with the series impedance *Z*, the joint impedance is expressed by Equation (2) if the impedance *Z* is inductance. Here, the complex impedance circle near the quartz resonant frequency is approximately two times smaller than when only one crystal is connected [24].

(2) $$\bar{Z_{2}}\left(\Omega \right)=\frac{\left(R_{1}\frac{1+j\frac{\omega_{0}L_{1}}{R_{1}}\left(\Omega -\frac{1}{\Omega }\right)}{1+\frac{C_{01}}{C_{1}}\left(1-\Omega^{2}\right)+j\frac{C_{01}}{C_{1}}\cdot \frac{R_{1}}{\omega_{0}L_{1}}\cdot \Omega }\right)\left(R_{2}\frac{1+j\frac{\omega_{0}L_{2}}{R_{2}}\left(\Omega -\frac{1}{\Omega }\right)}{1+\frac{C_{02}}{C_{2}}\left(1-\Omega^{2}\right)+j\frac{C_{02}}{C_{2}}\cdot \frac{R_{2}}{\omega_{0}L_{2}}\cdot \Omega }\right)}{\left(R_{1}\frac{1+j\frac{\omega_{0}L_{1}}{R_{1}}\left(\Omega -\frac{1}{\Omega }\right)}{1+\frac{C_{01}}{C_{1}}\left(1-\Omega^{2}\right)+j\frac{C_{01}}{C_{1}}\cdot \frac{R_{1}}{\omega_{0}L_{1}}\cdot \Omega }\right)+\left(R_{2}\frac{1+j\frac{\omega_{0}L_{2}}{R_{2}}\left(\Omega -\frac{1}{\Omega }\right)}{1+\frac{C_{02}}{C_{2}}\left(1-\Omega^{2}\right)+j\frac{C_{02}}{C_{2}}\cdot \frac{R_{2}}{\omega_{0}L_{2}}\cdot \Omega }\right)}+j\frac{\Omega \cdot L_{L}}{\sqrt{L_{1}\cdot C_{1}}}+R_{L}$$

The series resonant frequency (without impedance *Z* connected in series) for two quartz crystals connected in parallel is approximately the same as for a single crystal and it is expressed by Equation (3).

(3) $$f_{0}\approx 1/\left(2\pi \cdot \sqrt{L_{1}\cdot C_{1}}\right)$$

In the state of switched Q (Figure 1) and connected switch A the oscillator’s frequency *f*_01q_ is calculated with Equation (4), where the impedance *Z* (inductance *L_L_*) is connected in series with the quartz crystal *Q*_1_ [14,21,22,23]:

(4) $$f_{01q}=f_{0}\cdot \left[1+\frac{C_{1}}{2\left(C_{01}+C_{p}-\frac{1}{\omega_{0}{}^{2}\cdot L_{L}}\right)}\right]\;$$

On the other hand, in the state of switched $\bar{Q}$, the oscillator’s frequency *f*_02q_ is determined with Equation (5), whereby the impedance *Z*_ref_ (inductance *L*_ref_) is now connected with the quartz crystal Q_1_ in series:

(5) $$f_{02q}=f_{0}\cdot \left[1+\frac{C_{1}}{2\left(C_{01}+C_{p}-\frac{1}{\omega_{0}{}^{2}\cdot L_{\mathrm{ref}}}\right)}\right]\;$$

When both switches A and B are on (Figure 1), the following two Equations (6) and (7) can be written, but the additional real capacitance *C*_02_ (Figure 2) of the second crystal has to be taken into account:

(6) $$f_{03qq}=f_{0}\cdot \left[1+\frac{C_{1}}{2\left(C_{01}+C_{02}+C_{p}-\frac{1}{\omega_{0}{}^{2}\cdot L_{L}}\right)}\right]$$

(7) $$f_{04qq}=f_{0}\cdot \left[1+\frac{C_{1}}{2\left(C_{01}+C_{02}+C_{p}-\frac{1}{\omega_{0}{}^{2}\cdot L_{\mathrm{ref}}}\right)}\right]$$

For both cases (single- or dual-quartz crystal units connected in parallel), Equations (8) and (9) for the pulling range between the switching of inductances *L*_L_ and *L*_ref_ can be written:

(8) $$Pulling\_range_{q}=\left(f_{01q}-f_{02q}\right)/f_{02q}$$

(9) $$Pulling\_range_{qq}=\left(f_{03qq}-f_{04qq}\right)/f_{04qq}$$

### 2.3. Experimental Inductance-to-Frequency Converter

**Figure 3:**
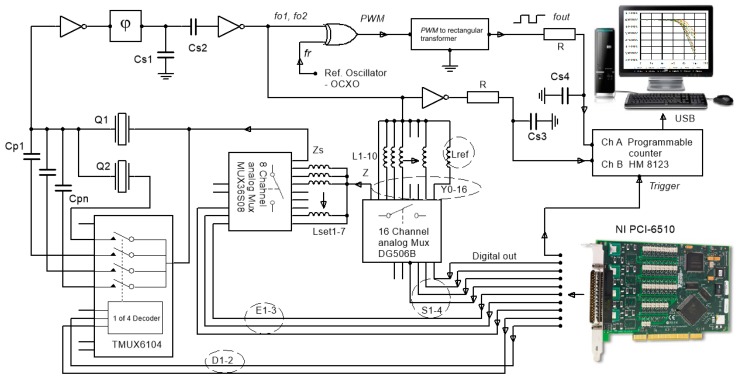
Automated inductance converter principle.

The quartz oscillator represents the main part of experimental converter. It works as a switching oscillator, in which at every switch, inductances (impedance Z) alternating between *L*_1_–*L*_10_, and reference inductance *L*_ref_ (*Z*_ref_) are changed in series with the quartz crystal *Q*_1_ (Figure 3). The problem to be solved is how to measure the inductance-to-frequency characteristics and at the same time achieve the reduction of any other influence, such as quartz crystal fundamental temperature characteristics, the influence of parasitic inductances, and capacitances to a minimum. By sequentially changing L_1_–L_10_ the oscillator’s frequency is changed in a given range close to the quartz crystal serial resonance frequency and the inductance-to-frequency characteristics of the converter can be measured. The aim of switching between the two inductances is to reduce the temperature influence of the quartz crystal fundamental frequency-temperature characteristics and the temperature dependence of inductance L_1_–L_10_ and *L*_ref_ (if it exists), because the quartz crystal and both of the inductances have the same temperature. The value of the frequency difference between the frequencies *f*_01_ and *f*_02_ depends on the value of L_1_–L_10_ and L_ref_. The initial setting of the frequencies *f*_01_ and *f*_02_ is carried out by setting the inductance L_set1-7_ by digital signals on the wires E_1_–E_3_ (Figure 3). 

The switching time between both frequencies can vary from a few milliseconds to a few seconds, and the switching procedure is carried out by I/O device NI PCI-6510 (National Instruments, UK & Ireland, Newbury) that controls 4, 8, and 16 channel analog multiplexers while using LabView (LV) software. With the help of the frequency *f*_r_ = 3.95 MHz (from the reference oven-controlled crystal oscillator (OCXO)), the XOR gate (which produces pulse width modulated (PWM) signal), and a signal transformer (which transforms the pulse-width modulated signal to the rectangular signal), the frequencies *f*_01_ and *f*_02_ in the range 3.8–3.95 MHz are converted to a low frequency range between 1 and 150 kHz (*f*_out_) [25,26]. The capacitances C_p1_–C_pn_ (Figure 3) are connected together with the four-channel low-capacitance analog multiplexer in parallel to the quartz crystal Q_1_ for the increase of the pulling sensitivity settings by the digital signals on the lines D_1_–D_2_ (increasing capacitance C_01_ or increasing C_01_ + C_02_, when both crystals together are connected in parallel (Figure 1 and Figure 3)). Using an eight-channel analog multiplexer and through an appropriate choice of inductance L_set1-7_, while taking into account *f*_r_ = 3.95 MHz and *f*_02_ (when inductance L_ref_ is connected), the output frequency in the range *f*_out_ = 1.9–5 kHz is set. It represents the initial output frequency signal of the converter. The frequencies *f*_out_, *f*_01_, and *f*_02_ are synchronously measured with HM 8123 programmable counter with regard to the switching frequency (*f*_Switch_ = 0.5–20 Hz). The LV software that controls the switching (lines S_1_–S_4_) of the frequency between *f*_01_ and *f*_02_, gathers the measurement data from the counter and processes it. The capacitance *C*_s1_ suppresses the spurious responses of the oscillator to avoid oscillation at higher frequencies. The *C*_s3_ and *C*_s4_ reduce the high frequency noise at the counter inputs [27]. The counter channel A measures the frequency difference *f*_out_ = *f*_01_ − *f*_r_ or *f*_02_ − *f*_r_, while channel B measures the frequencies *f*_01_ and *f*_02_, depending on the multiplexer switching table (S_1_–S_4_). The converter also enables the connection of a second crystal unit Q_2_ in parallel to the single-quartz crystal unit Q_1_ controlled by the multiplexer signals that are sent by wires D_1_–D_2_ to increase the frequency pulling.

### 2.4. The Principle of Converter’s Temperature and any Other Simultaneous Influence Compensation

In high-resolution inductance change measurement, the stable functioning of the converter in a given temperature range is crucial in reducing its influence, especially when dynamic temperature changes in the environment are involved. The principle of minimizing the influence of the temperature *T* and any other influence at same time, such as parasitic inductances and capacitances, is based on the switching of one of the inductance L_1_–L_10_ and reference inductance L_ref_, inductance L_set1-7_, positive feedback φ, and two inverters (Figure 3). In both cases of the switching inductances, the temperature influence on the change of the resonance frequency Δ*f*_0_ (*T*) of the quartz crystal is practically the same due to a millisecond or a second delay between the two switchings. The same applies to the influence of the quartz crystal ageing Δ*f*_0_ (*t*). Furthermore, the influence of the parasitic capacitances and inductances (which is also present in the electronic circuit itself) is, at both frequencies *f*_01_ and *f*_02_, also practically the same and reduced to a minimum after subtracting these two frequencies [28,29,30]. The pulse-width modulator (XOR gate) (Figure 3) was used to filter the frequency difference *f*_01_ − *f*_ref_ and *f*_02_ − *f*_ref_.

The converter output frequency *f*_out_ depends on the selection of digital signals D_1-2_, E_1-3_, and S_1-4_ (frequencies *f*_01_ and *f*_02_) and reference frequency *f*_r_ (Figure 3) and can be expanded to:

(10) $$\begin{array}{cc} f_{out}=[(f_{01}\left(L_{L}\right) & +\Delta f_{0}\left(T_{1}\right)+\Delta f_{0}\left(t_{1}\right))-(f_{r}\left(T_{1}\right)+\Delta f_{r}\left(T_{1}\right)+\Delta f_{c1}\left(t_{1}\right))]-[(f_{02}\left(L_{\mathrm{ref}}\right)+\Delta f_{0}\left(T_{2}\right)\\ & +\Delta f_{0}\left(t_{2}\right))-(f_{r}\left(T_{2}\right)+\Delta f_{r}\left(T_{2}\right)+\Delta f_{c2}\left(t_{2}\right))] \end{array}$$

In Equation (10), $\Delta f_{0}\left(T_{1}\right)$ and $\Delta f_{0}\left(T_{2}\right)$ represent the temperature influence on both oscillator frequencies $f_{01}$ and $f_{02}$, while $\Delta f_{r}\left(T_{1}\right)$ and $\Delta f_{r}\left(T_{2}\right)$ stand for the temperature influence on the reference oscillator at the two oscillator switchings. Since the frequency changes in Equation (10) between $\Delta f_{0}\left(T_{1}\right)$ and $\Delta f_{0}\left(T_{2}\right)$ are almost the same (as a result of the millisecond- or second-long time interval between the two switchings) due to the influence of the temperature, this influence is reduced to a minimum. Additionally, when the frequencies $\Delta f_{r}\left(T_{1}\right)$ and $\Delta f_{r}\left(T_{2}\right)$ of the reference oscillator change, the influence is reduced to a minimum since the influence of the temperature is approximately the same. In Equation (10) these two influences are subtracted due to the difference that is formed. Hence, it does not matter how big they, are since the switching between *f*_01_ and *f*_02_ is takes place in a short time period. The same also applies to the crystal ageing influence, i.e. the difference between $\Delta f_{0}\left(t_{1}\right)$ and $\Delta f_{0}\left(t_{2}\right)$. As this influence is practically the same within the millisecond time interval, it can also be ignored. To sum up, the switchings compensate for the temperature influence on both oscillator frequencies, the reference frequency, and crystal ageing, as shown by Equation (11).

(11) $$f_{out}=f_{01}\left(L_{L}\right)-f_{02}\left(L_{\mathrm{ref}}\right)+\Delta f_{c1}\left(t_{1}\right)-\Delta f_{c2}\left(t_{2}\right)$$

In Equation (11), $\Delta f_{c1}\left(t_{1}\right)$ and $\Delta f_{c2}\left(t_{2}\right)$ represent the counter errors [21,22], which are different at each switching and are difficult to define for the times *t*_1_ and *t*_2_ [27]. They include the oscillator noise (jitter, phase modulated, and thermal Johnson noise) and the counter measurement error. It is very difficult to differentiate between all of them. The frequency measurement errors of the frequencies *f*_01_ (L_L_), *f*_02_ (L_ref_), and *f*_out_ depend on the gate time (the longer the gate time, the lower frequency measurement error) of the HM 8123 counter (Hameg Instruments).

The frequencies *f*_out_, $f_{01}\left(L_{L}\right)$ and $f_{02}$($L_{\mathrm{ref}}$) are synchronously measured by the programmable counter (Figure 3) after every switching between two inductances. The LV software then calculates the frequency difference between the frequencies $f_{01}\left(L_{L}\right)$ and $f_{02}$($L_{\mathrm{ref}}$). The output frequency *f*_out_ depends almost uniquely on the inductance difference between the *L*_L_ and *L*_ref_, because all of the other influences are compensated (reduced to minimum). This means that Δ*f*_out_ (L_L_) frequency change is virtually independent of the quartz basic temperature characteristics Δ*f*_0_ (*T*), which represents a crucial novelty in converters. 

The switching principle has some limitations, despite the fact that it provides a considerable reduction of the influences, such as the temperature influence of elements, crystal ageing and the ageing of any other elements, reference oscillator’s instability, and the influence of the parasitic capacitances and inductances. There are basically two restrictions which are related to different times of the logical switchings of the two frequencies in the oscillator and the speed of the dynamic temperature change. The frequencies *f*_01_ (L_L_) and *f*_02_ (L_ref_) that are given by Equations (10) and (11) have different times *t*_1_ and *t*_2_ (one after another), and the subtraction result in Equations (10) and (11) is not exactly point-to-point in time performed. While taking into account the two frequency switchings in the oscillator, the measurements with the counter HM 8123 (~1 ms/channel), and the signal processing with the LV software ~3 ms (Figure 3), the converter’s response time is approximately 5 ms. Subsequently, if the temperature changes are sufficiently steep, then the temperature changes Δ*f*_0_ (*T*_1_) and Δ*f*_0_ (*T*_2_) in both Equations (10) and (11) are not equal, so they may not be cancelled. The dynamic frequency difference measurement error within ~2 ms (one measurement cycle for both switches) determines the maximum temperature variation (*ΔTmax/Δt*). The proposed method, first and foremost, considerably reduces the quartz crystal temperature influence, if the speed of the temperature change in not too big. 

## 3. Results

### 3.1. Experimental Data and Working Modes of the Converter

For this experiment, two AT-cut crystals with a frequency change of ±1 ppm that were in the temperature range *T* = 0–40 °C (Figure 3) were used. The data of the quartz (Q_1_, Q_2_) crystals’ (*f*_0_ = 4.075 MHz) equivalent circuit elements are L_1_ = 61.016 mH, C_1_ = 25 fF, R_1_ = 10 Ω, C_o_ = 4 pF, and quality Q_u_ = 153 k (measured by an HP4194A impedance analyzer (Hewlett Packard/Agilent). As a reference oscillator, an oven-controlled OCXO18T5S oscillator (Mercury) with a stability of ±0.01 ppm in the temperature range 0–40 °C was used with a warm-up time of approximately one minute [28,29,30,31,32]. The measurement probes (Ch A and Ch B) were connected to the converter frequency outputs (Figure 3). The LV software performed an algorithm in a sequence to control the switchings, to set inductances, read the frequency data from the counter, and process the data.

**Figure 4:**
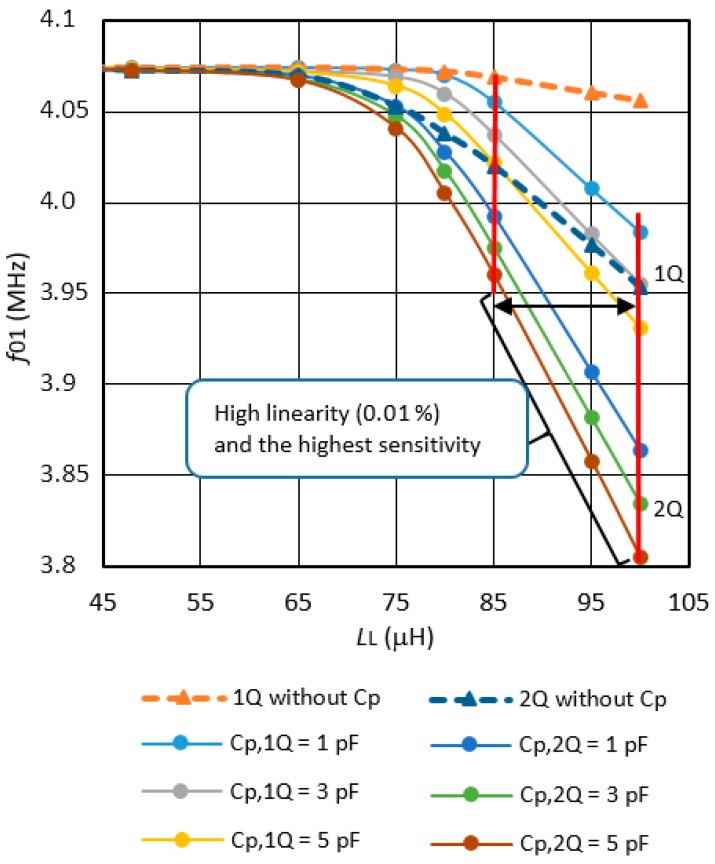
Inductance-to-frequency characteristics of the converter for inductance *L*_L_ settings in steps from 48.004 μH to 100.002 μH (without capacitance C_p_ – the dotted lines and for the different values of capacitance *C*_p_, and for the single- and dual-quartz units at *T* = 25 °C).

The experimental results show the comparison of the characteristics between the two working modes of the converter, i.e. when there is only single-quartz crystal unit (1Q) that was connected and when two crystals are connected in parallel (2Q). Moreover, the results for the additional working mode of the converter with parallel switchings of additional capacitors for both modes are shown. Additionally, the inductance-to-frequency converter characteristics for (1Q) and (2Q) at different sensitivities C_p_ are given (Figure 3 and Figure 4).

### 3.2. Inductance-to-Frequency Sensing Comparison with or without C_p_ for Single- and Dual-Quartz Crystal Units

The experimental results (Figure 4) show inductance-to-frequency sensing characteristics (*f*_01_) comparison for the single- and dual-quartz crystal units (1Q and 2Q) with regard to the change of the inductance L_L_ = L_1_ to *L*_10_ and the comparison of the characteristics either without C_p_ or for various values of C_p_ (Figure 1 and Figure 3) for the selection of different converter’s sensitivities while using a single- and dual-quartz crystal unit. 

The Inductance L_L_ values are set in steps of 48.004 μH, 65.001 μH, 75.005 μH, 80.003 μH, 85.002 μH, 90.003 μH, 95.001 μH, and 100.002 μH by a NI PCI-6510 device, analog multiplexers and LV software. The capacitors C_p_, inductances L_L_, and inductance L_ref_ were measured with a HP 4194A impedance analyzer with a tolerance of 0.1 %. Within the inductance range (L_L_ = 85–100 μH), the inductance-to-frequency characteristics is almost linear (0.01 %) (for 1Q—single quartz crystal and 2Q—dual quartz crystal). Table 1 shows a comparison of the single- and dual-quartz crystal unit’s sensitivity (0–40 °C) in the range L_L_ = 85–100 μH. 

It is hard to compare the sensitivity performance to other methods, because, the sensitivity is often expressed in mH/cm or in mV/mm in the voltage range 0–10 V or 0–3 V in other methods. The comparison of the resolution of proposed method to other methods (Colpitts circuit, Balance bridge circuit, and Phase circuit), however, shows that the proposed method has a resolution of 0.05 % with the temperature compensation, while the maximum resolution of other methods is 0.01 % in the same temperature range as the proposed method [18,19].

**Figure 5:**
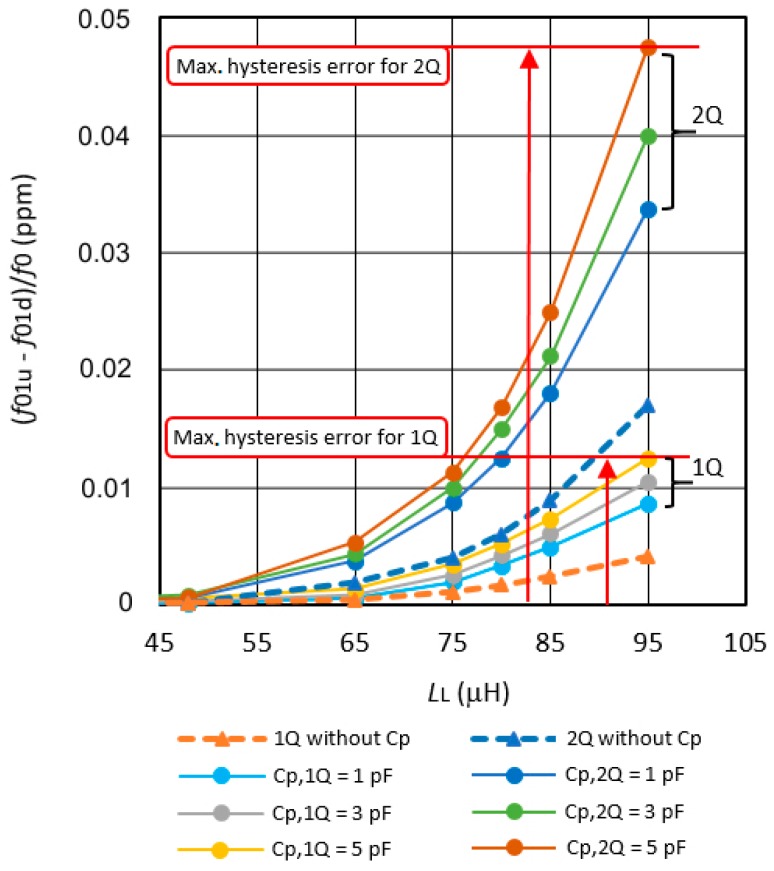
The relative frequency differences *f*_01u_ − *f*_01d_/*f*_0_ depending on the connection of the single- or dual-quartz crystal units in the oscillator without the capacitance C_p_ and for different capacitance values C_p_ = 1–5 pF.

Figure 5 shows the converter’s frequency hysteresis influence during the increase and decrease of the inductances L_L_ (Figure 4) and measurement of the frequency *f*_01_. A small difference in the frequency measurement that is not visible in Figure 4 can be detected. The relative frequency difference (*f*_01u_ − *f*_01d_) increases with increasing inductance L_L_ = L_1_ to L_10_.

The relative frequency differences *f*_01u_ − *f*_01d_/*f*_0_ for the same inductance settings if the inductance L_L_ = L_1_ to L_10_ is set (Figure 3) in steps of 48.004 μH, 65.001 μH, 75.005 μH, 80.003 μH, 85.002 μH, 90.003 μH, 95.001 μH to 100.002 μH (frequency *f*_01u_), and back from 100.002 μH to 48.004 μH in the same steps (frequency *f*_01d_) without C_p_ are presented, and for three different capacitances C_p_ = 1, 3, 5 pF for a single quartz crystal (1Q). In the same way, the results are shown for two quartz crystals (2Q) that are connected in parallel. In the output frequency *f*_out_ (Figure 3), the influences of the temperature, quartz ageing, and the influences of parasitic capacitances and parasitic inductances are reduced to a minimum, because, in the equation *f*_out_, the frequency difference ((*f*_01u_ − *f*_r_) − (*f*_02Lref_ − *f*_r_)) − ((*f*_01d_ − *f*_r_) − (*f*_02Lref_ − *f*_r_)) only remains (*f*_01u_ − *f*_01d_). The difference value (*f*_01u_ − *f*_01d_)/*f*_0_ in Figure 5 is increasing when the value of inductance L_L_ increases. In the range L_L_ = 85–95 μH, there is a maximum value of frequency difference at *L*_L_ = 95 μH and it is 0.048 ppm at the value of capacitance C_p_ = 5 pF and two quartz crystals that are connected in parallel (at the maximum sensitivity of the converter). 

### 3.3. Demonstration of the Dynamic Temperature Compensation of the Oscillator’s Frequency

To demonstrate the temperature compensation of the converter for single- and dual-quartz crystal, its electronic circuit was exposed to a dynamic temperature change in the range between 0–40 °C in the Weiss SB1 160 climate chamber (Weiss Umwelttechnik GmbH, Stuttgart, Germany). Due to the switching of the two inductances in the oscillator (Figure 3), i.e. between L_L_ and L_ref_, the temperature influence on the quartz crystal’s frequency in the oscillator is approximately the same (due to a few second delay between the two switches) at both of the switches and it has the same influence on both oscillator’s frequencies (*f*_01_ and *f*_02_). The temperature of the environment does not change much within this short time delay between the two switches. 

**Figure 6:**
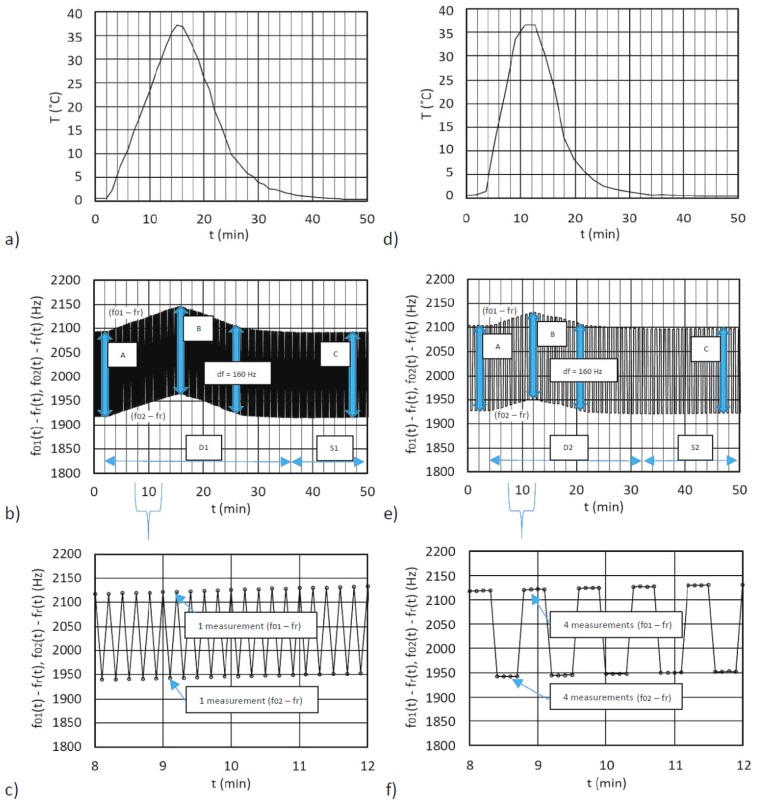
(**a**,**d**) Demonstration of the dynamic temperature change from 0–40 °C and back to 0 °C, (**b**,**e**) The change of the output frequency differences (*f*_01_(t) − *f*_r_(t)) and (*f*_02_(t) − *f*_r_(t)) during the temperature change, (**c**) The zoomed interval (Figure 6b) between eight to 12 minutes for a single measurement of the frequency difference (*f*_01_(t) − *f*_r_(t)) and (*f*_02_(t) − *f*_r_(t)) (**f**) The zoomed interval (Figure 6e) between 8 to 12 minutes for four measurements of the frequency difference (*f*_01_(t) − *f*_r_(t)) and (*f*_02_(t) − *f*_r_(t)).

The measurements were separately conducted for the single- and dual-quartz crystal units. Figure 6a illustrates the dynamic temperature change in the climate chamber (where the inductance converter was inserted) for a single-quartz crystal unit. The temperature was gradually changed from 0–40 °C and back to 0 °C. Figure 6b (range D1) shows the dynamical changes of the frequency differences *f*_01_(t) − *f*_r_(t) and *f*_02_(t) − *f*_r_(t) that result from the temperature influence. In the first experiment, only a single measurement was carried out as shown in the zoomed interval between eight and 12 minutes (Figure 6b,c). 

The frequency changes in the regions D1 and S1 are approximately the same, because the frequency differences A, B, and C (160 Hz) are almost the same when the inductances are fixed values L_01_ = 90.002 μH, L_02_ = 90.003 μH and L_ref_ = 90.004 μH, and capacitance is C_p_ = 5 pF. In the second experiment (Figure 6d–f)), the number of the frequency difference measurements was four (Figure 6f). The frequency changes in the regions D2 and S2 (the frequency differences A, B, and C) are approximately the same. Figure 6e demonstrates a very good temperature compensation across the whole dynamic temperature change from 0–40 °C and back to 0 °C (Figure 6d), which reduces the temperature influence to a minimum.

**Figure 7:**
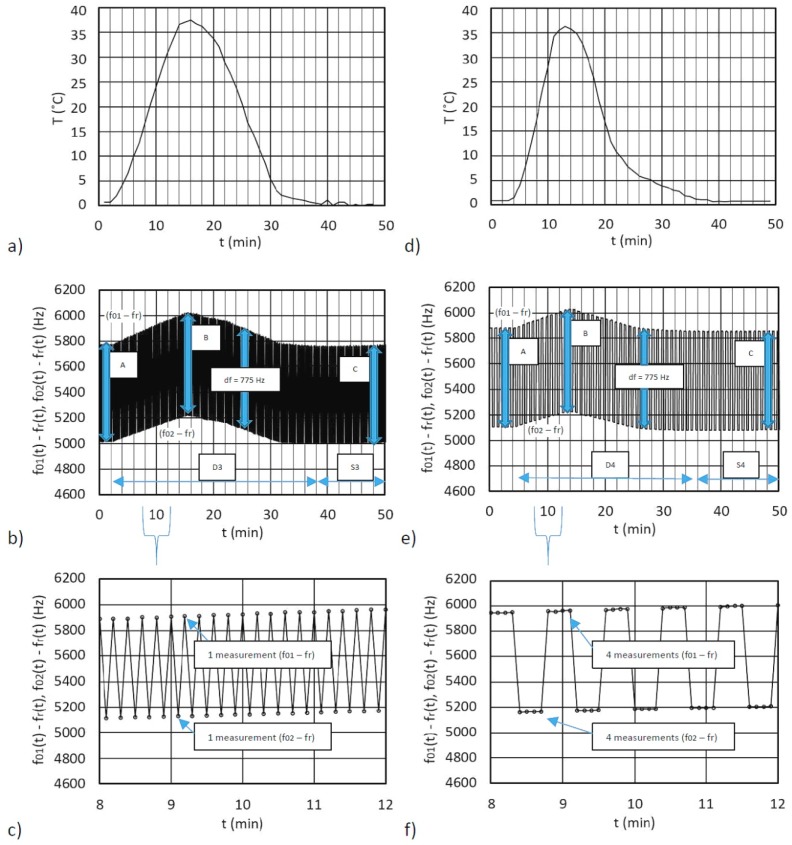
(**a**,**d**) Dynamic temperature change from 0 °C to 40 °C and back to 0 °C, (**b**,**e**) The change of the output frequency differences (*f*_01_(t) − *f*_r_(t)) and (*f*_02_(t) − *f*_r_(t)) during the temperature change, (**c**) The zoomed interval (Figure 7b) between eight and 12 minutes for one measurement of frequency differences (*f*_01_(t) − *f*_r_(t)) and (*f*_02_(t) − *f*_r_(t)) (**f**) The zoomed interval (Figure 7e) between 8 and 12 minutes for four measurements of frequency differences (*f*_01_(t) − *f*_r_(t)) and (*f*_02_(t) − *f*_r_(t)).

On the other hand, Figure 7a illustrates the dynamic temperature change in climate chamber for dual-quartz crystal unit in the inductance converter. The temperature was again gradually changed from 0–40 °C and back to 0 °C. 

Figure 7b (range D3) illustrates the dynamical changes of the frequency differences *f*_01_(t) − *f*_r_ (t) and *f*_02_(t) − *f*_r_(t) as a result of the temperature influence. This frequency difference is 775 Hz, and it is approximately the same (A, B and C) in the ranges D3 and S3 (at the same fixed inductances L_01_, L_02_, L_ref_, and the same C_p_, as used in Figure 6). In the first experiment, there was a single frequency measurement for each frequency, as shown in zoomed interval between eight and 12 minutes (Figure 7c). The frequency changes in the regions D3 and S3 display good temperature compensation of the oscillator’s frequencies, because the frequency differences A, B, and C are approximately equal. In the second experiment (Figure 7d–f)), the number of the frequency difference measurements was four (Figure 7f). The frequency difference changes in the regions D4 and S4 also display very good temperature compensation across the entire dynamical temperature change from 0–40 °C and back to 0 °C, which reduces the temperature influence to a minimum. 

The comparison of the converters with single- or dual-quartz crystal units shows that dual-quartz crystal (connected in parallel) converters work equally well (Figure 7). The size of the frequency differences between *f*_01_ − *f*_r_ (t) and *f*_02_ − *f*_r_ (t) (Figure 6b,e and Figure 7b,e) depend on the inductance difference between L_01-10_ and L_ref_ (for 1Q and 2Q the values were the same). Figure 6b and Figure 7b show that, for the same inductances L_01_, L_02_, L_ref_, and the same C_p_, the sensitivity was 4.5 times greater when dual-quartz crystal unit was used in the inductance converter. 

Table 2 shows the frequency difference *f*_out_ instability that occurs as a result of the dynamic temperature change (Figure 6a,d) and (Figure 7a,d) if the oscillator works with a single or two quartz crystals that are connected in parallel. The comparison of the frequency difference instability in both cases was performed under the same circumstances, i.e. when the C_p_, L_01_, L_02_, and L_ref_ were the same (oscillator’s working modes) for a single measurement and four of them. The error is greater if the time between the two switchings is longer or if the speed of the temperature change is faster. This error represents the dynamic changing of both frequency differences (*f*_01_ − *f*_r_) and (*f*_02_ − *f*_r_) as a result of the temperature change from 0–40 °C and back to 0 °C. It occurs at the output of the converter in a sequence when, after every two switches (one measurement cycle), both of the frequencies are subtracted, which represent the output signal *f*_out_. Table 2 compares the frequency difference instability Δ*f*_out_ for a single quartz crystal and two of them connected in parallel. The latter shows lower dynamic stability (Figure 7b) (range D3) when compared to Figure 6b (range D1) for a single quartz crystal and a single measurement. The dynamic stability (Figure 7e) (range D4, dual quartz crystals connected in parallel) as compared to Figure 6e (range D2) for single quartz crystal (four measurements) is also lower. A comparison of temperature-wise almost stable ranges when the temperature does not change so quickly anymore in Figure 6 and Figure 7 (Table 2 (S1 and S3 or S2 and S4)) and also shows lower instability when two quartz crystals are connected in parallel. 

All of the frequency difference instabilities are also presented as percentage (%). The dynamic frequency difference instability (comparing D1 and D2 or D3 and D4) is lower when there are four frequency measurements performed, because four measurements were made in sequence for each switch. Moreover, static frequency difference instability when the temperature does not change anymore (when comparing S1 and S2 or S3 and S4) turns out to be much smaller when as many as four measurements are performed. To sum up, if the temperature does not change so quickly, then the frequency difference instability (comparing S2 = 0.03% and S4 = 0.05%) is 1.7 times more; however, the inductance-to-frequency sensitivity is 4.5 times greater when the two quartz crystals connected in parallel are used instead of a single one. 

## 4. Discussion

The experimental results show that the automated procedures in the converter enable the measuring of the inductance-to-frequency characteristics with high precision, the setting of the initial output frequency *f*_out_, and the reduction of different influences, such as, for instance, temperature influence and the influence of parasitic impedances to a minimum. 

As demonstrated, the converter’s temperature compensation is successfully achieved with the method that switches between two inductances (the reference and the measuring one) in the quartz oscillator. In this way, the frequency instability of the oscillator resulting from the temperature characteristics (AT-cut) is compensated. The switching method’s efficiency depends upon the speed of the temperature change in the environment with time. It compensates both short-term as well as long-term temperature-frequency instability (ageing) of the quartz crystal, which means that the short-term instability occurs in the first few minutes of the converter’s operating, while the long-term instability (in high-quality quartzes) is determined with changes ±0.5 ppm (0–40 °C) through the years. The speed of the switching between two inductances impacts the temperature compensation, but it is reduced to a minimum due to the millisecond-long switching at dynamic environment temperature changes.

The increase of the inductance-to-frequency converter’s sensitivity is achieved through a parallel connection of the capacitance to a single crystal or to two of them. It is noteworthy that the parallel functioning of the two crystals further reduces the total impedance, the crystals oscillate in a stable way, more easily, and the frequency pulling is greater than with only one crystal and capacitance are connected in parallel. Additionally, in both cases, greater pulling can be achieved with greater parallel capacitance. When comparing to other oscillator types, the proposed ways of increasing the pulling sensitivity are well temperature-compensated and are less sensitive to parasitic inductances and capacitances. If the converter’s output frequency sensitivity of 41.5 kHz/μH (Table 1) is in the range L_L_ = 85–100 μH at C_P_ = 5 pF at the simultaneous connection of two quartz crystals, the supply voltage stability of the oscillator is 5 V ± 0.01 V, the counter accuracy is ±5 ⋅ 10^−9^ Hz (in the range from 0–40 °C), and the frequency reference *f*_r_ stability is ±0.01 ppm, then the output stability is Δ*f*_out_/*f*_0_ = ±0.05 ppm. This gives the inductance-to-frequency converter a resolution of ±1 pH in the temperature range of 0–40 °C. These experimental results promise great applicability of this automated switching method in the area of small inductance change measurement. 

## 5. Conclusions

In this paper, an enhanced inductance sensing performance of inductance-to-frequency converter using dual-quartz crystals was presented. The major strength of the proposed approach is the sensitivity improvement (sensitivity is 4.5 times greater) and simultaneous compensation procedure of the crystal’s natural temperature characteristics that are achieved through the switching method. It enables an additional reduction of the parasitic inductance and capacitance that are almost always present. The experimental results clearly show that the proposed inductance-to-frequency converter opens up new possibilities and ways of sensitivity improvement, parasitic influence reduction, ensuring, at the same time, the temperature compensation of the converter. The accuracy of the method is only limited by the speed of the switching between the reference and measuring inductance, the speed of the environment temperature change, and the frequency measurement error. 

This method can be applied in many measurements that require high sensitivity and resolution, where inductance plays an important role, i.e. low inductance measurement, impedance change measurement, and magnetic material properties measurement.

## Figures and Tables

**Table 1:** Converter’s sensitivity with C_p_ (pF) and without C_p_ for the single- and dual-quartz crystal units in parallel.

Sensitivity (kHz/μH)		
C_p_ (pF)	1Q	2Q
**without C_p_**	3.317	17.819
1	19.726	34.486
3	21.875	37.612
5	24.266	41.491

**Table 2:** Comparison of the frequency difference *f*_out_ for a single or two quartz crystals connected in parallel in the oscillator with the same *C*_p_ and the same inductance L_01_, L_02_, and L_ref_.

Oscillator’s Working Modes						
**Quartzes**		**1Q**			**2Q**	
*C*_p_ (pF)		3			3	
*L*_01_ (μH)		90.002			90.002	
*L*_02_ (μH)		90.003			90.003	
*L*_ref_ (μH)		90.004			90.004	
*f*_0ut_ = (*f*_01_ − *f*_r_) - (*f*_02_ − *f*_r_) (Hz)		160			775	
**Oscillator’s Frequency Difference Instability**						
**1Q**						
**Number of Measurements = 1 (Figure 6 and Figure 7)**						
Δ*f*_out_ (Hz) for Specific Ranges	D1 = ± 1.5		S1 = ± 0.30	D3 = ± 3		S3 = ± 2.1
Δ*f*_out_ (%) for Specific Ranges	D1 = ± 0.83		S1 = ± 0.18	D3 = ± 0.99		S3 = ± 0.27
**2Q**						
**Number of Measurements = 4 (Figure 6 and Figure 7)**						
Δ*f*_out_ (Hz) for Specific Ranges	D2 = ± 2.5		S2 = ± 0.05	D4 = ± 6.1		S4 = ± 0.4
Δ*f*_out_ (%) for Specific Ranges	D2 = ± 1.56		S2 = ± 0.03	D4 = ± 1.6		S4 = ± 0.05
